# Histological Analysis of the Lower-Positioned Transverse Ligament

**DOI:** 10.2174/1874364100701010017

**Published:** 2007-12-04

**Authors:** Takahashi Yasuhiro, Kakizaki Hirohiko, Kinoshita Shinsuke, Iwaki Masayoshi

**Affiliations:** 1 Department of Ophthalmology and Visual Sciences, Osaka City University Graduate School of Medicine. 1-4-3, Asahimachi, Abeno-ku, Osaka, 545-8585, Japan; 2 Department of Ophthalmology, Aichi Medical University. Nagakute, Aichi, 480-1195, Japan

## Abstract

The lower-positioned transverse ligament (LPTL) had been thought to run parallel to the junction between the orbital septum and the levator aponeurosis (junction). However, its true course was disclosed as crossing the junction. Since earlier histological studies were undertaken before the precise course was elucidated, it was uncertain whether the true LPTL was adequately disclosed. Therefore, we examined ten upper eyelids of 6 Asian patients who underwent blepharoptosis repairs. The LPTL and the tissue running parallel to the junction were harvested intraoperatively. Light-microscopically, the LPTL contained looser and thinner collagen bundles and less elastic fibres than the parallel tissue. Electron-microscopically, collagen microfibrils in the LPTL had almost the same periodicity and thickness as those in the parallel tissue. The LPTL is a loose and inelastic structure, which at a light microscopic level is completely different from the parallel tissue; however, the differences could not be verified by electron microscopy.

## INTRODUCTION

The lower-positioned transverse ligament (LPTL) is one of the main supporting structures of the upper eyelids [1,2], and usually exists in puffy upper eyelids [3]. Since the LPTL regulates upper eyelid height [3], when a thick LPTL occurs as in blepharoplasty or blepharoptosis surgery, incision of the LPTL is recommended to secure appropriate upper eyelid height [3].

**Fig. (1):**
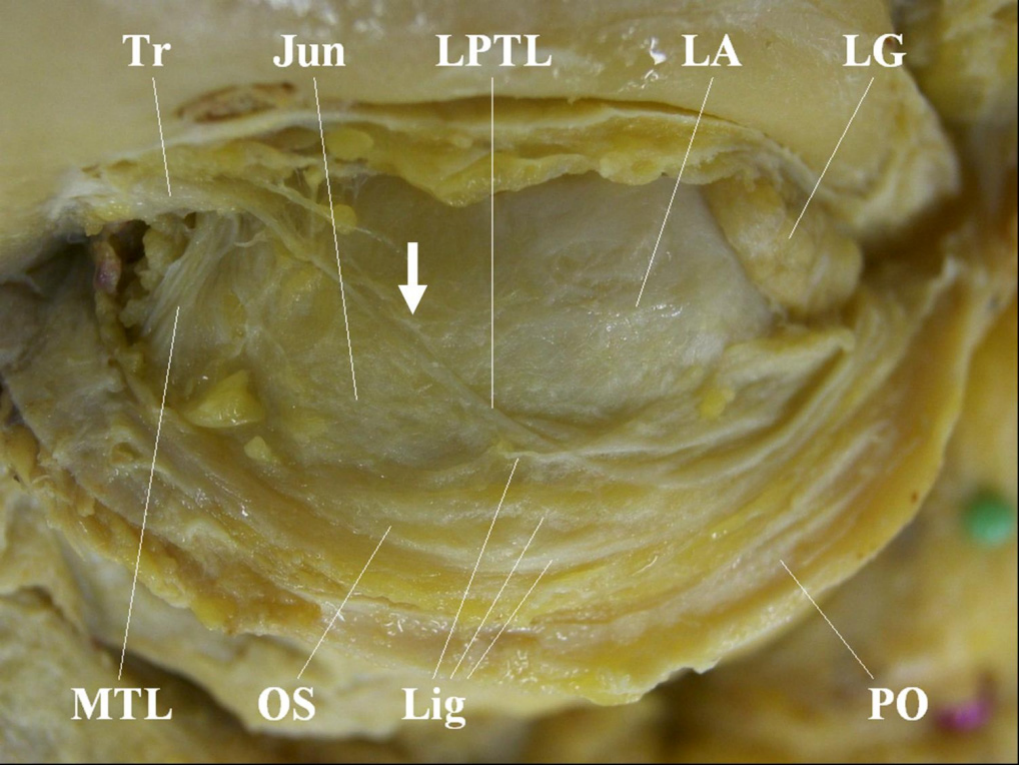
Modified course of the lower-positioned transverse ligament (LPTL) (top: superior; *left:* medial). Reprinted with permission of the *Japanese Journal of Ophthalmology* (Kakizaki H, *et al.* Posterior aspect of the orbital septum is reinforced by ligaments. *Jpn J Ophthalmol* 2005; 49: 477-80). The LPTL originates from the trochlea and runs in an inferolateral direction. The LPTL passes over the junction between the orbital septum and levator aponeurosis, attaches onto the posterior aspect of the orbital septum, and runs towards the lateral orbital rim. The LPTL was harvested in the part that the arrow indicates. *Tr*, trochlea; *Jun*, junction of the orbital septum and the levator aponeurosis; *LA*, levator aponeurosis; *LG*, lacrimal gland; *MTL*, medial horn tensing ligament; *OS*, orbital septum; *Lig*, ligaments on the posterior aspect of the orbital septum; *PO*, periosteum.

The LPTL was believed to be positioned around the lateral horn of the levator aponeurosis, and to run in parallel with a junction between the orbital septum and the levator aponeurosis (junction) [3]. However, some different anatomical findings on the LPTL have been reported [1,2]. The LPTL was shown to originate from the trochlea, run inferolaterally and to pass the junction, where it reflected the posterior aspect of the orbital septum, and finally to reach the lateral orbital rim (Fig. **1**). That is, the true LPTL differs from the tissue running parallel to the junction.

From histological findings, the LPTL was thought to contain tighter collagen bundles than the Whitnall ligament [3]. However, since the above noted histological results were obtained before elucidation of the precise course of the LPTL, it was uncertain whether the true LPTL was adequately harvested. As well, the LPTL has not been examined using transmission electron microscopy.

In the present study, we histologically examined the LPTL and the tissue running parallel to the junction using light and transmission electron microscopy.

## MATERIALS AND METHODOLOGY

Ten upper eyelids of 6 Asian patients (5 females and 1 male, average age 61.8 years old, range, 41-75), who underwent blepharoptosis repairs, were examined. Eight eyelids showed involutional blepharoptoses, and 2 blepharoptoses after cataract surgery. Patients with preceding traumas and/or past eyelid surgeries were excluded. All patients were informed of the study, and proper consent and approval were obtained preoperatively. All methods for securing human tissue were humane and complied with the tenets of the Declaration of Helsinki.

In blepharoptosis repairs, at first, the anterior surface of the tarsus was exposed. Dissection under the orbicularis oculi muscle was advanced superiorly to expose the junction. The orbital septum was transversely incised above the junction, because the transverse septal incision just on the junction sometimes complicates detection of the LPTL. Then, dissection was extended superomedially to confirm the course of the LPTL, which running on the aponeurosis was also confirmed to distinguish it from the Whitnall ligament [4,5]. We then harvested the LPTL in a part that ran inferolaterally immediately before crossing the junction. We also harvested the parallel tissue on the junction in the lower part in which the LPTL was harvested. Each of the tissues was divided into 2 parts; one was fixed in 10% buffered formalin and the other in 2% glutaraldehyde. Tissue sections of the formalin-fixed specimen were stained with Elastica van Gieson [6], which were also used in a former study [3], and examined light-microscopically (BX-40, OLYMPUS, Tokyo, Japan). The histological results we obtained were compared with those histological results presented earlier [3]. As well, ultra-slice sections fixed in glutaraldehyde were stained with lead and uranyl acetate and were examined with a transmission electron microscope (JEM-1200EX||, JEOL, Tokyo, Japan).

## RESULTS

**Fig. (2):**
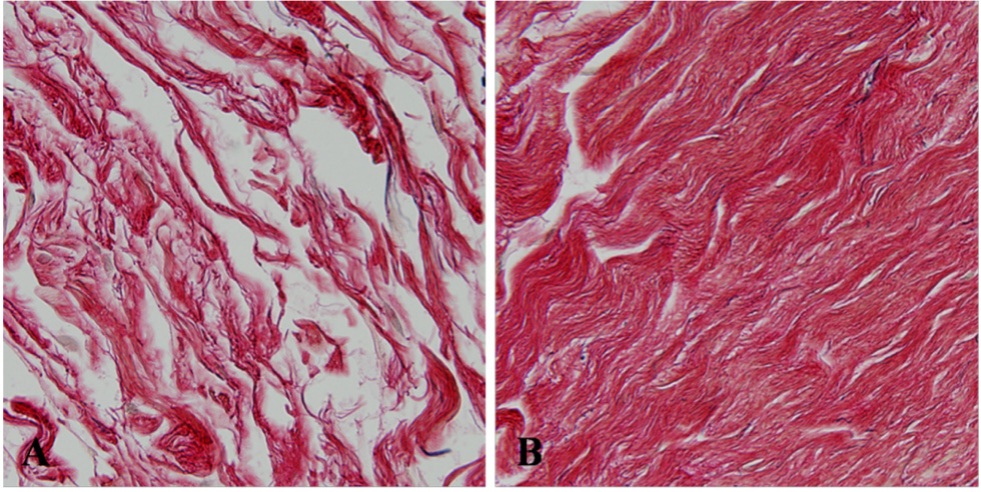
Light microscopic findings. **(A)** Lower-positioned transverse ligament. Collagen bundles are scattered and thin. **(B)** The parallel tissue on the junction between the orbital septum and the levator aponeurosis. Thick collagen bundles with a few elastic fibres congregate.

**Fig. (3):**
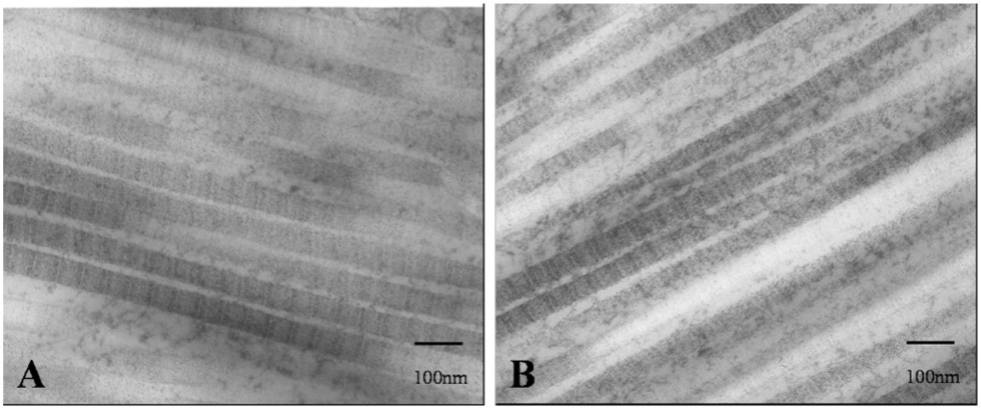
Transmission electron microscopic findings. **(A)** Lower-positioned transverse ligament. **(B)** The parallel tissue on the junction between the orbital septum and the aponeurosis. Both tissues contain microfibrils with the same periodicity and thickness.

Light-microscopically, the LPTL in all cases contained looser and thinner collagen bundles with less elastic fibres than the tissue running parallel to the junction (Fig. **2A**). The LPTL was histologically analogous to the previously reported Whitnall ligament [3], while the tissue on the junction (Fig. 2B) was analogous to the previously reported LPTL [3]. Electron microscopic findings showed that collagen microfibrils of the LPTL (Fig. 3A) had almost the same periodicity and thickness as those in the parallel tissue on the junction (Fig. 3B).

## DISCUSSION

The LPTL showed a different light-microscopic composition to that of the tissue running parallel to the junction, although both tissue architectures could not be distinguished electron microscopically, indicating that the strength of each tissue was different. Each tissue could have specific physiological functions depending on their anatomical position. Since the LPTL contains looser and thinner collagen bundles and less elastic fibres than the parallel tissue, the strength loaded onto the LPTL might be less than that on the parallel tissue. Thus, most upper eyelids, except ones with a very robust LPTL [2,3], can move appropriately without restriction by the LPTL. Also, the tighter and thicker composition of the parallel tissue is reasonably suitable for repetitional eye blinking because the junction provides a pivotal position to lift the preaponeurotic fat pad [7].

The LPTL reinforces the orbital septum, with which it sustains a preaponeurotic fat pad [1]. Especially in Asians, a supporting structure is necessary for the orbital septum because preaponeurotic fat tends to drop inferiorly and project anteriorly due to puffiness. This is why the LPTL is a desirable structure to support the preaponeurotic fat pad. However, the smaller elasticity of the LPTL sometimes causes a problem in which the extension of the levator aponeurosis is disturbed. Ordinary thin collagen bundles of the LPTL may be an adaptation to compensate for this drawback. Since the LPTL runs obliquely [1], roughly coinciding with the direction of blinking, the LPTL needs to be incised in cases with insufficient eyelid closure in blepharoptosis repairs.

The LPTL shares some common macroscopic features with Whitnall ligament [8], indicating that both structures may have derived from the same origin [8,9]. Although the present study did not histologically examine the Whitnall ligament, compared to light-microscopic findings of the Whitnall ligament [3], the LPTL showed histological similarities. In this, the present study largely supports the hypothesis that both ligaments share the same origin.

## CONCLUSION

The LPTL is a loose and inelastic structure, which is a different structure from that of the tissue running parallel to the junction, but showed similarities to the Whitnall ligament. Although there may be some limitations in the present study in terms of blepharoptoses and age-related degenerative changes, we believe that the results here will help towards a precise understanding of the anatomy of the upper eyelid.

## References

[1] Kakizaki H, Zako M, Miyaishi O (2005). Posterior aspect of the orbital septum is reinforced by ligaments. Jpn J Ophthalmol.

[2] Kakizaki H, Zako M, Nakano T (2005). Modified course of the lower-positioned transverse ligament. Br J Plast Surg.

[3] Yuzuriha S, Matsuo K, Kushima H (2000). An anatomical structure, which results in puffiness of the upper eyelid and a narrow palpebral fissure in the Mongoloid eye. Br J Plast Surg.

[4] Anderson RL, Dixon RS (1979). The role of Whitnall's ligament in ptosis surgery. Arch Ophthalmol.

[5] Ettl A, Zonneveld F, Daxer A, Koornneef L (1998). Is Whitnall's ligament responsible for the curved course of the levator palpebral superioris muscle?. Ophthalmic Res.

[6] Stevens A, Lowe J (2005). support cells and the extracellular matrix. Human Histology.

[7] Kakizaki H, Zako M, Nakano T (2005). The levator aponeurosis consists of two layers that include smooth muscle. Ophthal Plast Reconstr Surg.

[8] Kakizaki H, Zako M, Nakano T (2004). Anatomical study of the lower-positioned transverse ligament. Br J Plast Surg.

[9] Hwang K, Kim DJ, Chung RS, Lee SI, Hiraga Y (1998). An anatomical study of the junction of the orbital septum and the levator aponeurosis in Orientals. Br J Plast Surg.

